# For Better or Worse: The Effect of Prismatic Adaptation on Auditory Neglect

**DOI:** 10.1155/2017/8721240

**Published:** 2017-09-12

**Authors:** Isabel Tissieres, Mona Elamly, Stephanie Clarke, Sonia Crottaz-Herbette

**Affiliations:** Neuropsychology and Neurorehabilitation Service, Centre Hospitalier Universitaire Vaudois (CHUV) and University of Lausanne, Lausanne, Switzerland

## Abstract

Patients with auditory neglect attend less to auditory stimuli on their left and/or make systematic directional errors when indicating sound positions. Rightward prismatic adaptation (R-PA) was repeatedly shown to alleviate symptoms of visuospatial neglect and once to restore partially spatial bias in dichotic listening. It is currently unknown whether R-PA affects only this ear-related symptom or also other aspects of auditory neglect. We have investigated the effect of R-PA on left ear extinction in dichotic listening, space-related inattention assessed by diotic listening, and directional errors in auditory localization in patients with auditory neglect. The most striking effect of R-PA was the alleviation of left ear extinction in dichotic listening, which occurred in half of the patients with initial deficit. In contrast to nonresponders, their lesions spared the right dorsal attentional system and posterior temporal cortex. The beneficial effect of R-PA on an ear-related performance contrasted with detrimental effects on diotic listening and auditory localization. The former can be parsimoniously explained by the SHD-VAS model (shift in hemispheric dominance within the ventral attentional system; Clarke and Crottaz-Herbette 2016), which is based on the R-PA-induced shift of the right-dominant ventral attentional system to the left hemisphere. The negative effects in space-related tasks may be due to the complex nature of auditory space encoding at a cortical level.

## 1. Introduction

Unilateral spatial neglect tends to include distinct auditory deficits, which are often referred to as auditory neglect and are investigated with a variety of experimental paradigms [1]. The key feature of auditory neglect, impaired attention to left-sided stimuli, has been initially revealed in tasks of *dichotic listening*. In this paradigm, simultaneous auditory stimuli are presented to either ear; extinction or significant decrease in reporting stimuli presented to the left ear has been considered as a manifestation of auditory neglect [2, 3]. Although often present in auditory neglect, left ear extinction on dichotic listening has been also reported in two conditions which are unrelated to neglect. Left ear extinction is a key feature of the callosal disconnection syndrome [4, 5] and is associated with lesions of the splenium and isthmus of the corpus callosum [6, 7]. Furthermore, contralateral ear extinction has been reported to occur as often after left as right hemispheric lesions, when the damage extended to auditory-related structures [8]. The ambiguity in the interpretation of left ear extinction as a sign of auditory neglect has led to the introduction of the *diotic listening paradigm*, which consists of two simultaneous stimuli presented to the right or left by means of interaural time differences. Extinction or significant decrease in reporting stimuli lateralized to the left and/or bilateral decrease in reported stimuli is a characteristic of the right hemispheric lesions and depends critically on the integrity of basal ganglia [9–11]. *Auditory mislocalization* and in particular systematic directional errors to the ipsilesional side are believed to be another manifestation of auditory neglect [12–14]. Particularly striking symptom is *alloacusis*, that is, the misplacement of auditory stimuli across the midline. The three key features of auditory neglect, left-sided extinction on dichotic or diotic listening, and the distortion of auditory space perception can occur independently of each other and involve distinct neural networks; very likely, they correspond to different types of auditory neglect [9–11]. The three key features of auditory neglect are often associated with visuospatial neglect symptoms [1, 10, 11, 14], which are treated with different approaches, including prismatic adaptation [15–22].

Prismatic adaptation has gained much interest, partly because of its well-documented effect on visuospatial neglect [15–22]. It consists of a visuomotor task during which the subject points to visual targets while wearing glasses mounted with right-deviating prisms. After an initial phase, when the subject overshoots the targets to the right, the pointing becomes correct. After the removal of the prisms, the first trials show pointing errors to the left, referred to as the aftereffect [20]. A series of neuroimaging studies was carried out in normal subjects to investigate neural mechanisms underlying the effect of R-PA on visual attention. The stages of visuomotor adaptation were shown to involve the posterior parietal cortex and the cerebellum on the right side [23–27]. An overall effect of a brief exposure to R-PA is the change of visuospatial representations in the inferior parietal lobule (IPL) in both hemispheres. As demonstrated in a recent study, the representation of the left, center, and right visual fields is enhanced in the left IPL and the representation of the right visual field decreased in the right IPL [28]. Thus, R-PA appears to shift the right-dominant ventral attentional system to the left hemisphere; in neglect, this shift is likely to restore the alerting input to the dorsal attentional system on either side and contribute thus to the alleviation of attentional deficits in visuospatial neglect [29].

Several lines of evidence suggest that visual and auditory attention relies on a supramodal attentional network. Activation studies have shown that in the context of spatial and nonspatial attentional tasks visual and auditory stimuli involve the same cortical regions and hence most likely a shared attentional network [30–33]. Similarly, the frequent cooccurrence of visual and auditory attentional deficits in unilateral neglect was proposed to reflect the supramodal nature of the syndrome [1, 34]. Further support comes from two studies which reported that R-PA alleviates specific symptoms of auditory neglect. A first study focused on the effect of R-PA on dichotic listening and reported in a group of 6 patients an alleviation of left ear extinction on dichotic listening, without affecting general arousal [35]. A second study investigated the effect of R-PA on spatial gradients in visual and auditory target detection and described in a group of 12 patients an overall improvement of auditory target detection, without restoring the spatial gradient of attention [36]. It is currently unknown whether R-PA affects other symptoms of auditory neglect.

The effect of R-PA on specific symptoms of auditory neglect may rely on the shift of the right-dominant ventral attentional system to the left IPL, as postulated in the SHD-VAS model for visuospatial attention [37]. If so, the alleviation of auditory neglect symptoms would depend on the integrity of the right dorsal attentional system and its access to the left IPL. We have investigated how R-PA affects key features of auditory neglect, namely performance on dichotic and diotic listening and auditory localization, and what the underlying anatomical constraints are. We hypothetized that the restoration of the alerting input from ventral attentional system via the left IPL may alleviate auditory neglect symptoms if the remaining parts of the involved network are intact. Thus, we postulated that for the effect of R-PA to occur, the dorsal attentional system (within the right hemisphere) and the afferent interhemispheric pathway from the left IPL need to be intact. We have expected that these mechanisms are likely to play a role in dichotic and diotic listening tasks. We did not expect a systematic improvement of sound localization performance, because of the great complexity in auditory space encoding (for detailed description see discussion [38–40]).

## 2. Methods

### 2.1. Participants

**Figure 1:**
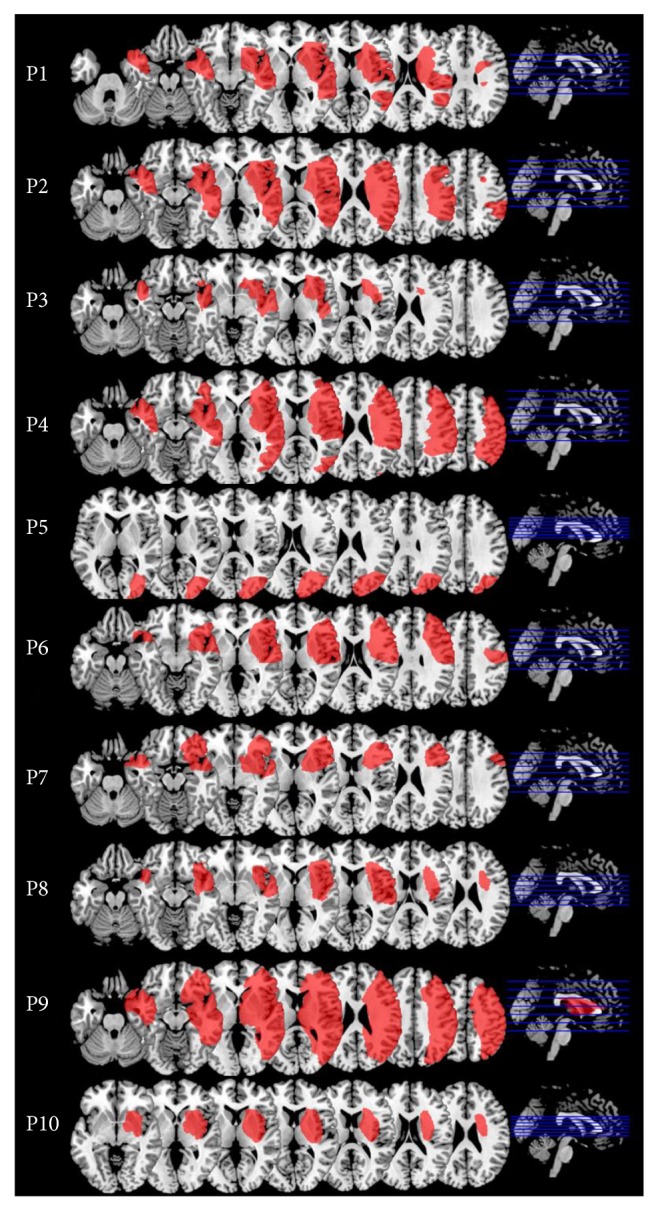
Lesions of individual patients displayed on axial slices of a normalized MRI template (positions of the slices in blue).

Ten consecutive stroke patients with unilateral spatial neglect and without history of psychiatric or previous neurological affections participated in this study (6 men, mean age 59.6 years ± 7.1; Table 1). The inclusion criteria were (i) a first unilateral right hemispheric ischemic stroke; (ii) normal or corrected to normal visual acuity, compatible with performing visual tasks without prescription glasses (so that prisms can be worn); and (iii) normal hearing thresholds at a tonal audiometry and less than 12 dB difference between the ears (average across all frequencies). All patients sustained an ischemic infarction in the territory of the right middle cerebral artery (Figure 1) and presented at the time of testing visuospatial and auditory neglect. The mean delay between the R-PA and the stroke was 95 days ± 34. The patients were recruited among the patients of the Neuropsychology and Neurorehabilitation clinic of the Lausanne University Hospital (CHUV), and all provided an informed consent. Seventeen normal subjects served as control population for comparing the aftereffect in the ecological R-PA paradigm used here with the aftereffect observed in a shorter version R-PA used in a previous study (8 men, mean age 26.5 years ± 3.6; [28]). The study was conducted in accordance with the Declaration of Helsinki (1964) and was approved by the Ethic Committee of the Canton de Vaud, Switzerland.

### 2.2. Prismatic Adaptation

The ecological R-PA paradigm involved an adaptation phase during which the subject wore prisms which deviated the entire visual field 10° to the right (as in previous studies [17, 18, 20, 29, 41, 42]). The adaptation phase lasted 30 minutes during which the subject carried out a sequence of six different visuomotor activities, three of which resulted in sound production: (i) playing a sequence of 3 tones on a colour-coded xylophone according to the colours on a card shown by the experimenter; (ii) ringing 3 coloured bells in a sequence chosen from a group of 7 according to the colours on a card shown by the experimenter; (iii) placing five cups according to the pattern shown by the experimenter; (iv) picking up one bell identified by its colour among seven bells and ringing it; (v) placing a token in a column (among five) which the experimenter designated by its number (Puissance4® game); and (vi) placing Scrabble® tokens in the correct order to form three-letter words presented visually by the experimenter. Each activity lasted 5 minutes. The movements during these activities are slower than simple pointing movements in the classical adaptation; to reach the total number of movements which was shown to be critical for maximal adaptation to occur [43], we increased the duration of the adaptation phase to 30 minutes.

The aftereffect of R-PA, that is, visuomotor pointing error which occurs during the first pointing after the removal of the prisms, was assessed as in the previous studies [17, 18, 20, 37, 41, 42]. Briefly, the subject's head was positioned on chinrest and two black dots placed at a distance of 57 cm 14° to the left or to the right of his body midline; the proximal two-thirds of the distance between the subject and the dots were hidden. When positioned in the apparatus, the subject was asked to look at one of the dots, close his eyes, and point to the dot; this procedure was repeated twice for each dot. The aftereffect was expressed in degrees, corresponding to the average of the four measures. All patients performed the ecological R-PA paradigm, and all but one (P6) were tested for visuo-pointing errors before and after R-PA. P6 was not able to perform the aftereffect measure because he could not maintain the eyes closed during the pointing.

### 2.3. Evaluation of Auditory Neglect

#### 2.3.1. Dichotic Listening Task

The dichotic listening task consisted of thirty pairs of disyllabic words presented simultaneously, one word to the left and another to the right ear (same paradigm as in [9, 11, 44]). The subjects were instructed to be attentive to both ears and to report both words. Performance was assessed by the total number of correct responses for each ear and by the lateralization index (right minus left ear, divided by right plus left ear, multiplied by 100). The performance of a control population was published previously [9]. The mean number of items reported for the right ear stimuli was 29.2 (SD = 1.685) and for the left ear stimuli 28.85 (SD = 2.74); the number of items reported for the left versus right ears did not differ significantly (*p* = 0.1004). The mean lateralization index was 0.986 (SD = 4.45).

#### 2.3.2. Diotic Listening Task

The diotic listening task consisted of thirty pairs of words presented simultaneously. Both words were presented at the same intensity level to both ears, but one was lateralized to the right hemispace and the other one to the left hemispace, using interaural time difference of 1 ms (same paradigm as in [9, 11, 44]). For both tasks, performance was assessed by the total number of correct responses for each side separately and by the lateralization index (right minus left side, divided by right plus left side, multiplied by 100). The performance of a control population was published previously [9]. The mean number of items reported for the right space was 26.15 (SD = 4.632) and for the left space 24.867 (SD = 5.02). There was a significant advantage for the right space (*p* = 0.0001). The mean lateralization index was 3.521 (SD = 5.96).

#### 2.3.3. Auditory Localization Task

The auditory localization task comprised 60 stimuli which were lateralized with interaural time differences (same paradigm as in [9, 11, 44–51]. The stimuli were bumblebee sounds, ranging from 20 to 10,000 Hz presented during 2 s including 100 ms rising and falling times. Five different azimuthal positions (12 sounds at each position) were simulated by interaural time differences (ITD), creating one central (no ITD) and four lateral positions, two in each hemispace. For the lateral positions, the ITD was 0.3 ms or 1 ms. The task consisted in indicating precisely the perceived position of the blumblebee on a graduated semicircle affixed on the headphone (from 0° at the vertex to 90° at each ear) with the right index finger. The overall performance of auditory localization was assessed by the relative positions attributed to two consecutive stimuli (global score). Responses were counted as correct when the position of the sound was indicated to the left or to the right of the previous stimulus in agreement with the difference in ITD or within ±10° of the previous location for identical stimuli; the maximal number of correct responses was 59. To quantify directional bias, more specific measures were used: (i) the number and the direction of alloacusis and (ii) the discrimination between neighbouring positions, by means of *t*-test between reported positions of nearby lateralizations (LL versus L; R versus RR). The performance of a control population was published previously [9, 44]. The mean global score was 57.15 (SD = 1.79). The mean for the central stimulus was −0.09° (SD = 4.5°). The mean index of response bias was 0.00 (SD = 0.74). Control subjects never exhibited alloacusis. Ten percent of control subjects failed to discriminate the two positions within one hemispace, never within both hemispaces.

### 2.4. Evaluation of Visuospatial Neglect

All patients were assessed for visuospatial aspects of neglect using the bells test and the line bisection task (“Batterie d'évaluation de la négligence spatiale” (BEN)) [52], as well as the evaluation of unilateral extinction for visual and tactile stimuli, search for neglect symptoms in visual target detection, graphical production, and motor performance (as in [9–11, 44, 50, 51]).

### 2.5. Statistical Analysis of Behavioural Data

Behavioural data from the dichotic and diotic tasks were tested for normality using the Shapiro-Wilk normality test, and due to the nonnormality of the distribution, the effect of R-PA was evaluated at the group level by a repeated measures nonparametric *F*-test. This method, used in a previous study [53], is a bootstrapping of the subjects (with replacement) and permutation of the within-subject factors. An *F* value is calculated on each cycle, for each randomization. Repeating this for 1000 cycles generates an empirical distribution of *F* values from which a corresponding *p* value is obtained. These analyses were processed using Python (Python Software Foundation, https://www.python.org/). For the dichotic listening task factors, ear (left, right) and session (pre- and post-R-PA) were used, for diotic listening side (left, right) and session (pre- and post-R-PA).

### 2.6. Lesion Analysis

Lesions were outlined on MRI (*n* = 4) or CT scan (*n* = 6) anatomical sequences using the Medical Imaging Interaction Toolkit (MITK) software (http://mitk.org). The superposition of the lesions was calculated using Statistical Parametric Mapping (SPM12, Wellcome Department of Cognitive Neurology, London, UK).

## 3. Results

### 3.1. Rightward Prismatic Adaptation and Its Aftereffects

The visuomotor effect of R-PA was evaluated by the presence of the aftereffect, that is, leftward deviation in pointing immediately after prism removal. Seventeen control subjects performed the ecological R-PA paradigm; their mean aftereffect was −8.55° (SD = 2.61°), which is within the range of aftereffects obtained with a shorter version of the R-PA paradigm in a previous study [28]. All but one patient (P6) were able to perform the pointing measures before and after R-PA, and all presented the expected leftward shift. The mean aftereffect was −5.88° (SD = 3.28°).

### 3.2. Dichotic Listening

The effect of R-PA was evaluated at a group level by a repeated measures nonparametric *F*-test [53]. The number of items reported for either ear yielded a significant main effect of ear (*F* (1, 9) = 11.12, *p* = 0.002) and a significant main effect of session (*F* (1, 9) = 5.13, *p* = 0.023), but only a trend for the interaction (*F* (1, 9) = 3.36, *p* = 0.056). The lateralization index did not differ significantly between pre- and post-R-PA (Wilcoxon signed-rank test, *Z* = −1.481, *p* = 0.139).

At an individual level, we have identified 8 patients who had a significant decrease of the left ear reporting and an abnormal lateralization index prior to R-PA (Table 2). After R-PA, 4 patients (P3, P5, P6, and P8) normalized their performance on dichotic listening, both in terms of items reported for the left ear and lateralization index. Four other patients (P1, P2, P4, and P9) did not improve their performance and presented after R-PA a significant decrease of left ear reporting and abnormal lateralization index.

The patients who responded to R-PA versus those who did not differ in terms of the site and extent of their lesion. The nonresponders tended to have larger lesions (range: 135.4–383.0 cm^3^; Table 1) than responders (range: 19.7–118.7 cm^3^). In nonresponders, but not in responders, the lesions extended over large parts of the temporo-parietofrontal cortex and the underlying white matter, including the superior parietal lobule, the intraparietal sulcus, and the posterior part of the temporal lobe. The patients who had normal performance in dichotic listening before R-PA (P7 and P10) had a relatively small lesion (38.1 and 70.6 cm^3^), which largely spared the temporoparietal cortex.

In summary, R-PA had a striking effect on left ear extinction in dichotic listening in some but not all patients with initial deficit. In responders, the superior parietal lobule, the intraparietal sulcus, and the posterior part of the temporal lobe tended to be spared, but not in nonresponders.

### 3.3. Diotic Listening

The effect of R-PA was evaluated at a group level by a repeated measures nonparametric *F*-test [53]. The number of items reported for either side yielded a significant main effect of side (*F* (1, 9) = 9.95, *p* = 0.006) and a significant main effect of session (*F* (1, 9) = 7.93, *p* = 0.014), but no significant interaction (*F* (1, 9) = 0.94, *p* = 0.375). The lateralization index did not differ significantly between pre- and post-R-PA (Wilcoxon signed-rank test, *Z* = −0.652, *p* = 0.515).

At an individual level, we have identified one patient (P1) who had a significant decrease of reporting for both the right and left spaces prior to R-PA, albeit with a lateralization index within the normal range (Table 2). After R-PA, this patient normalized his reporting for the right space, but remained deficient for the left space; the lateralization index was then outside the normal range, favouring the right space. Another patient (P4), who had a normal performance in diotic listening, including a normal lateralization index, prior to R-PA, increased after R-PA reporting for the right but not the left space; his lateralization index was then outside the normal range, favouring the right space. The two patients in whom R-PA induced a rightward spatial bias (P1 and P4) did have rather large lesions (135.4 and 202.6 cm^3^; Table 1) which extended over large parts of the temporoparietofrontal cortex and the underlying white matter.

The remaining 8 patients had right and left space reporting as well as lateralization index within the normal range before and after R-PA. Among them, three had pre-R-PA scores for the right and/or left side reporting in the lower range (P5; P8 and P9). After R-PA, two of them (P5 and P8) increased considerably both scores, whereas the third one (P9) did not. The former two (P5 and P8) sustained rather small lesions (19.7 and 44.1 cm^3^; Table 1) which spared the superior parietal lobule, the intraparietal sulcus, and basal ganglia. The latter one (P9) sustained a large lesion (382.0 cm^3^), which extended over large parts of the hemisphere and included the superior parietal lobule, the intraparietal sulcus, and basal ganglia.

In summary, R-PA induced in specific cases rightward spatial bias in diotic listening by enhancing the reporting within the right but not the left space. This profile was associated with extended lesions which included the superior parietal lobule, the intraparietal sulcus, and basal ganglia. In a few cases, R-PA improved the left side reporting from low to high normal range. The integrity of the superior parietal lobule, the intraparietal sulcus, and basal ganglia appeared to be essential for this to occur.

### 3.4. Auditory Localization

At a group level, there was no statistically significant difference between pre- and post-R-PA global score measures (*Z* = −1.19, *p* = 0.234) nor for the number of left-to-right (*Z* = −1.461, *p* = 0.144) or right-to-left alloacusis (Z = 0, *p* = 1; for all comparisons, Wilcoxon signed-rank test).

Prior to R-PA, all patients were deficient at one or several of the following scores: (i) global score; (ii) the location attributed to the central stimulus; (iii) discriminating L-LL plus R-RR; and (iv) presence of alloacusis (Table 3). After the exposure to R-PA, only one patient (P2) improved his performance and reached normal range. His lesion was rather large (182.5 cm^3^; Table 1) and extended over large parts of the temporoparietofrontal cortex and the underlying white matter.

The remaining nine patients worsened their performance. Three (P1, P4, and P9) enhanced their rightward bias by shifting the position attributed to the central stimulus to the right and/or by increasing the number of left-to-right alloacusis, thus aggravating neglect symptoms. Their lesions were rather large (135.4 and 382.0 cm^3^) and extended over large parts of the temporoparietofrontal cortex and the underlying white matter. Two patients (P3 and P7) became deficient on their global score, without increasing a rightward bias. Their lesions were relatively small (93.1 and 70.6 cm^3^) and extended over the anterior and posterior temporal lobes, frontal convexity, and/or the underlying white matter. One patient (P10) sustained leftward bias by shifting the position attributed to the central stimulus to the left and failed to discriminate the L-LL positions. Her lesion was relatively small (38.1 cm^3^) and subcortical.

In summary, the effect of R-PA on auditory localization was varied and in nine of ten cases detrimental. In specific cases, R-PA induced rightward spatial bias in auditory localization. There did not seem to be clear relationship between the site of lesion and the effect of R-PA on auditory localization.

## 4. Discussion

### 4.1. Alleviation of Auditory Neglect by Prismatic Adaptation: Ear versus Space

The most striking effect of R-PA which we have observed was the alleviation of left ear extinction on dichotic listening, present in half of the patients. This beneficial effect on ear-related performance contrasted with the modest or even detrimental effects on space-related measures. In diotic listening, we observed an improvement which was limited to reporting the right-space stimuli and created thus rightward spatial bias. In a few cases, R-PA had mostly negative effect on auditory localization, leading to a rightward spatial bias.

The diverging effects of R-PA on different aspects of auditory neglect may be partially explained by the underlying mechanisms. Whereas, the effect on dichotic listening is likely to depend on the same neural mechanisms as the effect on visuospatial attention, the complexity of the encoding of the auditory space at a cortical level may interfere with the effect on auditory localization and possibly on diotic listening.

### 4.2. Neural Mechanisms Underlying the Effect of Prismatic Adaptation in Auditory Neglect

Visual attention and orienting have been shown to depend on the dorsal and ventral attentional systems. As demonstrated in a series of seminal studies, the dorsal attentional network, which comprises the superior parietal lobule, the intraparietal sulcus, and the superior frontal cortex of both hemispheres, mediates endogenous allocation of visuospatial attention [54]. Its key region, the intraparietal sulcus, encodes predominantly the contralateral visual space [55]. Exogenous attention, that is, the alerting targets that appear at unattended locations, is mediated by the ventral attentional network, which is lateralized to the right hemisphere and includes the temporoparietal junction, IPL, and posterior part of the superior temporal gyrus; this region receives visual information from the whole visual space [54]. The right-dominant ventral and the bilateral dorsal attentional systems are interconnected, so that the alerting input from the ventral system can activate the dorsal system [56]. There is a reciprocal interconnection between the right and left parts of the dorsal attention system [56–58], characterized by an asymmetrical inhibitory effect by which the right posterior parietal cortex inhibits the left homologous region [57, 58].

A brief exposure to R-PA was shown to shift the right-dominant ventral attentional system to the left IPL. The task used in this study was the detection of visual target presented in the left, central, and right spaces, known to activate the ventral attentional system. R-PA leads to a significant increase of the ipsilateral visual field representation in the left IPL and a significant decrease in the right IPL [28]. This same study demonstrated that R-PA did not have the same effect on other types of visuospatial processing, such as visuospatial working memory. In a later study, the shift of the ventral attentional system from the right to the left hemisphere was demonstrated with the same visual detection task in neglect patients [29]. The model derived from these studies, referred to as SHD-VAS (shift in hemispheric dominance within the ventral attentional system), offers a parsimonious explanation for the effects of R-PA on visuospatial attention in normal subjects and neglect patients (for discussion see [37]). This model may be also relevant for auditoryspatial attention, since the dorsal and the ventral attentional systems are involved in auditory attention. Early activation studies reported that auditory alertness involved an extended right hemispheric network, including frontal, cingular, inferior parietal, temporal, and thalamic regions [32] and shared with visual alertness a common region within the ventral attentional system [33].

In view of the above quoted evidence, it is reasonable to assume that the effect of R-PA on auditory neglect relies on the shift of the right-dominant ventral attentional system to the left hemisphere. For the beneficial effect on attentional orienting to the left, the ventral attentional system within the left hemisphere needs to access the dorsal attentional system within the right hemisphere. Thus, a spared dorsal attentional system and intact inputs from the left IPL are necessary for such beneficial effects.

### 4.3. Effect of Prismatic Adaptation on Dichotic Listening: What Matters?

**Figure 2:**
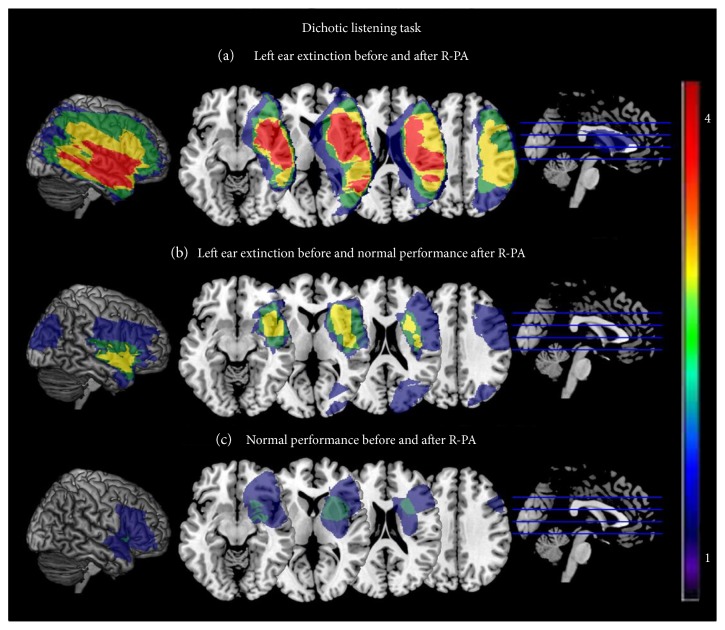
Anatomical correlates of performance in dichotic listening task. Superposition of lesions associated with 3 profiles: (a) Patients with left ear extinction who did not respond to R-PA (P1, P2, P4, and P9). (b) Patients with left ear extinction who responded to R-PA (P3, P5, P6, and P8). (c) Patients without deficits at the dichotic listening task (P7 and P10).

In our population, R-PA alleviated left ear extinction in dichotic listening in four patients, while it failed to do so in four others. The prerequisite for the beneficial effect of R-PA appeared to be intact with the superior parietal lobule, posterior part of the temporal lobe, as well as the periventricular white matter, which convey fibers joining the middle and posterior parts of the corpus callosum (Figure 2). The key role of the superior parietal lobule and of the callosal connections is in agreement with the SHD-VAS model.

Left ear extinction on dichotic listening has been also reported independently of the neglect syndrome, in cases of callosal disconnection and in particular when the splenium and the isthmus of the corpus callosum were damaged [6, 7]. These posterior parts of the corpus callosum are known to convey fibers from the temporal lobe, whereas the parietal callosal pathway tends to involve more anterior parts [59]. In our patient population, we did not have lesions which damaged specifically either the auditory or the parietal callosal pathway. Thus, it remains unclear whether R-PA would alleviate left ear extinction in cases with focal lesions of the splenium and the isthmus, that is, without damage to the dorsal attentional system and the more anterior callosal pathway.

### 4.4. Worsening Rightward Bias on Diotic Listening

Our results suggest that in specific conditions, R-PA can enhance rightward spatial bias and thus amplify neglect symptoms. When it happened in diotic listening, the initial condition involved scores that were pathologically low or within lower normal range on both sides. R-PA increased the reporting on the right but not on the left side. The beneficial effect on the right side reporting can be explained by the SHD-VAS model and the ensuing activation of the left dorsal attentional system. Both patients who presented this effect (P1 and P4) sustained damage to the right dorsal attentional system, which precluded reorienting attention to the left.

R-PA can enhance the left side reporting in diotic listening, as observed in two patients whose scores were in the lower normal range prior to R-PA and in the upper normal range after it (P5 and P8). Both patients had intact dorsal attentional system on the right side.

### 4.5. Disturbing Auditory Localization

Our results on auditory localization demonstrate that R-PA can enhance rightward spatial bias and thus aggravate neglect symptoms. Three patients presented this profile (P1, P4, and P9); after prismatic adaptation, they shifted the central position to the right and/or presented more right-to-left alloacusis. All three sustained damage to the right dorsal attentional system, which may explain the paradoxical rightward bias.

Apart from the enhancement of rightward spatial bias, R-PA tended to deteriorate more generally performance in auditory localization and even introduced a pathological leftward spatial bias. The former was observed in two patients whose global score became deficient after R-PA (P3 and P7), the latter in two other patients with a leftward spatial bias for the central position after R-PA (P5 and P10). These varied and rather unfavourable effects of R-PA on auditory localization may be related to the way auditory space is represented at a cortical level. Several lines of evidence indicate that auditory space is not represented in a topographical fashion, but encoded within specific neuronal populations [60–62]. Single neurons in nonhuman primates were reported to have large receptive fields, centered on the contralateral space [62–64]. Human fMRI studies reported a similar organization with preferential responses to contralateral locations and broad spatial tuning [38, 39]. The representation of the auditory space in humans appears to be lateralized, with greater bilaterality in the right and stricter contralaterality in the left hemisphere [40]. This asymmetry is particularly striking within the parietofrontal cortex, as demonstrated in activation [65–68], magnetoencephalography [69], transcranial magnetic stimulation [70, 71], and lesion studies [44]. This frontoparietal asymmetry is further supported by the patterns of structural and functional connectivities [72, 73].

The above quoted evidence suggests that the region invested by the ventral attentional system, and in particular the IPL, not only supports auditory alertness and attention, but also the representation of auditory space. When shifted to the left hemisphere after R-PA, the ventral attentional system most likely upkeeps its alerting function, and hence the positive effect on dichotic listening, as reported previously [35] and here. The representation of the auditory space, which depends on fine-tuned interactions within neuronal populations, is very likely disturbed by the exposure to R-PA. This may account for the detrimental effect of R-PA on sound localization.

## 5. Conclusions

The beneficial effect of R-PA on auditory neglect appears to be limited to the alleviation of left ear extinction in dichotic listening. This particular effect can be parsimoniously explained by the SHD-VAS model, that is, shift in hemispheric dominance within the ventral attentional system, induced by R-PA. This model has been initially formulated on the basis of visual activation studies [28, 29], but its predictions appear to be valid for the effect of R-PA on left ear extinction in dichotic listening. In particular, the observation that the right dorsal attentional system needs to be intact to obtain an alleviation of left extinction after R-PA is entirely in adequation with this model. This observation is clinically relevant, since it identifies anatomical profiles of patients for whom R-PA is likely to alleviate ear-related symptoms of auditory neglect.

The effect of R-PA on space-related measures of auditory neglect is varied and mostly detrimental. This is particularly apparent in auditory localization and may be accounted for by the complex way auditory space is represented at a cortical level. Whether the exacerbation of auditory localization deficits after exposure to R-PA has an impact on activities of daily living is currently not known. The effect may be short lived and possibly rapidly corrected as previously described for the realignment of visuo- and auditoryspatial representations in the ventriloquism effect [74–76].

## Figures and Tables

**Table 1:** Patients' characteristics including the delay between the stroke and the testing session. STG: superior temporal gyrus; MTG: middle temporal gyrus; IFG: inferior frontal gyrus; IPL: inferior parietal lobule; SMG: supramarginal gyrus; AG: angular gyrus; SPL: superior parietal lobule; ITG: inferior temporal gyrus; HG: Heschl gyrus; TTG: transverse temporal gyrus; GP: globus pallidus; SFG: superior frontal gyrus.

Patient	Sex	Age	Handedness	Neurological and neuropsychological deficits	Regions involved in lesion	Delay (days)	Lesion vol (cm^3^)
P1	M	53	Right	Left hemisyndrome (upper and lower limbs), multimodal neglect, nonspatial attentional deficits, executive dysfunction	STG, MTG, insula, IFG, temporal pole, putamen, caudate, precentral	54	135.4
P2	M	59	Right	Left unilateral homonymous hemianopia, severe multimodal neglect, executive dysfunction	STG, MTG, precentral, postcentral, IPL, IFG, insula, SMG, temporal pole, putamen, MFG, AG	80	182.6
P3	F	64	Right	Mild multimodal neglect and nonspatial attentional deficits	Insula, STG, temporal pole, MTG, putamen, IFG, caudate	59	93.1
P4	M	51	Left	Left hemisyndrome (upper and lower limbs), multimodal neglect, visuospatial apraxia, deficits in working memory and calculation, executive dysfunction	MFG, IFG, MTG, STG, precentral, postcentral, insula, SMG, temporal pole, occipital, putamen, precuneus, AG, SPL, ITG, HG, TTG, caudate	154	202.6
P5	M	57	Right	Horner syndrome on the right side, left unilateral homonymous hemianopia, severe multimodal neglect, nonspatial attentional deficits, deficit in anterograde episodic memory, executive dysfunction	Middle occipital, cuneus, superior occipital, MTG, cuneus, precuneus, AG, calarine	121	19.7
P6	M	59	Right	Left hemisyndrome (predominantly upper limb), left unilateral homonymous hemianopia, multimodal neglect, nonspatial attentional deficits, visuospatial apraxia, deficit in anterograde episodic memory, executive dysfunction	IFG, MFG, STG, precentral, insula, putamen, postcentral, temporal pole, precentral, MTG	89	118.7
P7	F	69	Right	Severe visuospatial neglect, nonspatial attentional deficits, mild executive dysfunction	IFG, MFG, STG, insula, putamen, temporal pole, MTG	122	70.6
P8	F	73	Right	Multimodal neglect, visuospatial apraxia, deficit in anterograde episodic memory, executive dysfunction	Insula, STG, IFG, putamen, MTG, HG, TTG	60	44.1
P9	M	58	Right	Left hemisyndrome (upper and lower limbs), severe multimodal neglect, deficit in anterograde episodic memory, executive dysfunction	Insula, putamen, caudate, GP, thalamus	127	382.0
P10	F	53	Right	Visuospatial neglect, nonspatial attentional deficits	MFG, STG, IFG, MTG, IPL, insula, postcentral, precentral, SMG, AG, precuneus, putamen, caudate, temporal pole, thalamus, hippocampus, parahippocampal gyrus, SFG	84	38.1

**Table 2:** Performance in dichotic and diotic listening tasks before (pre-R-PA) and after (post-R-PA) exposure to R-PA, listing the number of words reported for the left and right ears as well as the lateralization index. Scores outside the normal range are highlighted in bold.

	Dichotic listening task						Diotic listening task					
	Pre-R-PA			Post-R-PA			Pre-R-PA			Post-R-PA		
Patient	Left	Right	Lat. index	Left	Right	Lat. index	Left	Right	Lat. index	Left	Right	Lat. index
P1	**1**	30	**93.6**	**4**	29	**75.8**	**10**	**13**	13.0	15	26	**26.8**
P2	**3**	29	**81.3**	**4**	30	**76.5**	22	21	−2.3	20	22	4.8
P3	**19**	29	**20.8**	29	29	0.0	25	26	2.0	27	28	1.8
P4	**15**	27	**28.6**	**15**	27	**28.6**	18	24	14.3	16	29	**28.9**
P5	**19**	29	**20.8**	25	30	9.1	19	24	11.6	29	28	−1.8
P6	**22**	28	**12.0**	26	29	5.5	23	27	8.0	24	26	4.0
P7	29	30	1.7	30	30	0.0	25	27	3.9	28	30	3.5
P8	**20**	30	**20**	26	30	7.1	20	19	−2.6	24	27	5.9
P9	**7**	30	**62.2**	**2**	30	**87.5**	17	20	5.6	17	19	5.6
P10	29	28	−1.8	30	29	−1.7	26	27	1.9	28	29	1.8

**Table 3:** Performance in auditory localization before (pre-R-PA) and after (post-R-PA) exposure to R-PA. Scores outside the normal range are in bold. The global score corresponds to the number of stimuli correctly placed to the left or the right of the previous stimulus. The perceived positions of each of the five stimulus locations are indicated in degrees (positive in the right, negative in the left space). The ability to discriminate between the two positions within either hemispace (LL versus L; R versus RR) was assessed by *t*-tests; positions which failed to be discriminated are highlighted in bold. In the control population, 10% of subjects failed to discriminate the two positions within one hemispace, never within both hemispaces. The number of alloacusis is indicated separately for those where stimuli presented on the left were indicated on the right (L to R) and those where stimuli presented on the right were indicated on the left (R to L). Control subjects never presented alloacusis.

	Pre-R-PA								Post-R-PA							
		Positions (°)					Alloacusis			Positions (°)					Alloacusis	
Patient	Global score	LL	L	CE	R	RR	L to R	R to L	Global score	LL	L	CE	R	RR	L to R	R to L
P1	**51**	**−57.5**	**−53.8**	−2.1	**44.2**	**59.6**	0	0	**47**	**−43.6**	**−35.5**	−3.5	**29.6**	**49.6**	**2**	**2**
P2	55	−82.5	−66.3	**11.9**	55.8	75.0	0	0	57	**−76.3**	**−70.8**	7.5	49.6	70.8	0	0
P3	56	**−30.4**	**−28.3**	**−23.3**	36.7	43.3	0	0	**54**	−32.5	−20.8	**29.6**	**35.0**	**39.6**	0	0
P4	**42**	**−12.1**	**−14.5**	−5.6	**32.2**	**13.0**	**4**	**5**	**42**	**−9.5**	**0.5**	5.0	**28.0**	**27.3**	**7**	**1**
P5	56	**−46.3**	**−41.3**	**−9.2**	**40.0**	**43.8**	0	0	55	−68.3	−52.1	**−26.7**	26.7	47.9	0	0
P6	54	**−40.4**	**−36.7**	**−10.4**	28.3	44.2	0	0	59	**−37.1**	**−36.3**	**−15.4**	22.5	32.9	0	0
P7	54	−59.5	3.5	**41.3**	**58.9**	**68.0**	**4**	0	**46**	**−27.9**	**−28.8**	**17.0**	**29.4**	**60.0**	**3**	0
P8	**52**	−50.8	−37.1	**−24.6**	42.1	57.1	0	0	53	−67.1	−64.2	**−32.1**	**64.2**	**77.9**	0	0
P9	54	**−73.3**	**−68.3**	−0.8	**57.5**	**67.1**	0	0	**44**	**21.7**	**−30.9**	**40.8**	**56.7**	**76.3**	**11**	**1**
P10	**52**	−60.8	−64.2	7.5	**71.3**	**72.3**	0	0	**50**	**−75.0**	**−73.8**	**−48.5**	**82.7**	**80.4**	0	0

## Abbreviations

IPL: Inferior parietal lobule
R-PA: Rightward prismatic adaptation
SHD-VAS: Shift in hemispheric dominance within the ventral attentional system.

## References

[1] Pavani F., Husain M., Ládavas E., Driver J. (2004). Auditory deficits in visuospatial neglect patients. *Cortex; a Journal Devoted to the Study of the Nervous System and Behavior*.

[2] Heilman K. M., Valenstein E. (1972). Auditory neglect in man. *Archives of Neurology*.

[3] Hugdahl K., Wester K., Asbjørnsen A. (1991). Auditory neglect after right frontal lobe and right pulvinar thalamic lesions. *Brain and Language*.

[4] Kimura D. (1967). Functional asymmetry of the brain in dichotic listening. *Cortex*.

[5] Sparks R., Geschwind N. (1968). Dichotic listening in man after section of neocortical commissures. *Cortex*.

[6] Pollmann S., Maertens M., Yves D., Lepsien J., Hugdahl K. (2002). Dichotic listening in patients with splenial and nonsplenial callosal lesions. *Neuropsychology*.

[7] Sugishita M., Otomo K., Yamazaki K., Shimizu H., Yoshioka M., Shinohara A. (1995). Dichotic listening in patients with partial section of the corpus callosum. *Brain*.

[8] Renzi E. D., Gentilini M., Pattacini F. (1984). Auditory extinction following hemisphere damage. *Neuropsychologia*.

[9] Bellmann A., Meuli R., Clarke S. (2001). Two types of auditory neglect. *Brain: A Journal of Neurology*.

[10] Spierer L., Meuli R., Clarke S. (2007). Extinction of auditory stimuli in hemineglect: space versus ear. *Neuropsychologia*.

[11] Thiran A. B., Clarke S. (2003). Preserved use of spatial cues for sound segregation in a case of spatial deafness. *Neuropsychologia*.

[12] Bisiach E., Cornacchia L., Sterzi R., Vallar G. (1984). Disorders of perceived auditory lateralization after lesions of the right hemisphere. *Brain: A Journal of Neurology*.

[13] Haeske-Dewick H., Canavan A. G. M., Hömberg V. (1996). Sound localization in egocentric space following hemispheric lesions. *Neuropsychologia*.

[14] Soroker N., Calamaro N., Glicksohn J., Myslobodsky M. S. (1997). Auditory inattention in right-hemisphere-damaged patients with and without visual neglect. *Neuropsychologia*.

[15] Farnè A., Rossetti Y., Toniolo S., Làdavas E. (2002). Ameliorating neglect with prism adaptation: visuo-manual and visuo-verbal measures. *Neuropsychologia*.

[16] Frassinetti F., Angeli V., Meneghello F., Avanzi S., Làdavas E. (2002). Long-lasting amelioration of visuospatial neglect by prism adaptation. *Brain*.

[17] Pisella L., Rode G., Farnè A., Tilikete C., Rossetti Y. (2006). Prism adaptation in the rehabilitation of patients with visuo-spatial cognitive disorders. *Current Opinion in Neurology*.

[18] Rode G., Klos T., Courtois-Jacquin S., Rossetti Y., Pisella L. (2006). Neglect and prism adaptation: a new therapeutic tool for spatial cognition disorders. *Restorative Neurology and Neuroscience*.

[19] Rode G., Rossetti Y., Boisson D. (2001). Prism adaptation improves representational neglect. *Neuropsychologia*.

[20] Rossetti Y., Rode G., Pisella L. (1998). Prism adaptation to a rightward optical deviation rehabilitates left hemispatial neglect. *Nature*.

[21] Serino A., Bonifazi S., Pierfederici L., Làdavas E. (2007). Neglect treatment by prism adaptation: what recovers and for how long. *Neuropsychological Rehabilitation*.

[22] Serino A., Angeli V., Frassinetti F., Làdavas E. (2006). Mechanisms underlying neglect recovery after prism adaptation. *Neuropsychologia*.

[23] Chapman H. L., Eramudugolla R., Gavrilescu M. (2010). Neural mechanisms underlying spatial realignment during adaptation to optical wedge prisms. *Neuropsychologia*.

[24] Clower D. M., Hoffman J. M., Votaw J. R., Faber T. L., Woods R. P., Alexander G. E. (1996). Role of posterior parietal cortex in the recalibration of visually guided reaching. *Nature*.

[25] Danckert J., Ferber S., Goodale M. A. (2008). Direct effects of prismatic lenses on visuomotor control: an event-related functional MRI study. *The European Journal of Neuroscience*.

[26] Küper M., Wünnemann M. J. S., Thürling M. (2014). Activation of the cerebellar cortex and the dentate nucleus in a prism adaptation fMRI study. *Human Brain Mapping*.

[27] Luauté J., Schwartz S., Rossetti Y. (2009). Dynamic changes in brain activity during prism adaptation. *The Journal of Neuroscience: The Official Journal of the Society for Neuroscience*.

[28] Crottaz-Herbette S., Fornari E., Clarke S. (2014). Prismatic adaptation changes visuospatial representation in the inferior parietal lobule. *Journal of Neuroscience*.

[29] Crottaz-Herbette S., Fornari E., Notter M. P., Bindschaedler C., Manzoni L., Clarke S. (2017). Reshaping the brain after stroke: the effect of prismatic adaptation in patients with right brain damage. *Neuropsychologia*.

[30] Salo E., Rinne T., Salonen O., Alho K. (2013). Brain activity during auditory and visual phonological, spatial and simple discrimination tasks. *Brain Research*.

[31] Smith D. V., Davis B., Niu K. (2010). Spatial attention evokes similar activation patterns for visual and auditory stimuli. *Journal of Cognitive Neuroscience*.

[32] Sturm W., Longoni F., Fimm B. (2004). Network for auditory intrinsic alertness: a PET study. *Neuropsychologia*.

[33] Thiel C. M., Fink G. R. (2007). Visual and auditory alertness: modality-specific and supramodal neural mechanisms and their modulation by nicotine. *Journal of Neurophysiology*.

[34] Rode G., Pagliari C., Huchon L., Rossetti Y., Pisella L. (2017). Semiology of neglect: an update. *Annals of Physical and Rehabilitation Medicine*.

[35] Jacquin-Courtois S., Rode G., Pavani F. (2010). Effect of prism adaptation on left dichotic listening deficit in neglect patients: glasses to hear better?. *Brain*.

[36] Eramudugolla R., Boyce A., Irvine D. R. F., Mattingley J. B. (2010). Effects of prismatic adaptation on spatial gradients in unilateral neglect: a comparison of visual and auditory target detection with central attentional load. *Neuropsychologia*.

[37] Clarke S., Crottaz-Herbette S. (2016). Modulation of visual attention by prismatic adaptation. *Neuropsychologia*.

[38] Derey K., Valente G., de Gelder B., Formisano E. (2016). Opponent coding of sound location (azimuth) in planum temporale is robust to sound-level variations. *Cerebral Cortex (New York, N.Y.: 1991)*.

[39] McLaughlin S. A., Higgins N. C., Stecker G. C. (2016). Tuning to binaural cues in human auditory cortex. *Journal of the Association for Research in Otolaryngology: JARO*.

[40] Stecker G. C., McLaughlin S. A., Higgins N. C. (2015). Monaural and binaural contributions to interaural-level-difference sensitivity in human auditory cortex. *NeuroImage*.

[41] Jacquin-Courtois S., O’Shea J., Luauté J. (2013). Rehabilitation of spatial neglect by prism adaptation: a peculiar expansion of sensorimotor after-effects to spatial cognition. *Neuroscience & Biobehavioral Reviews*.

[42] Redding G. M., Rossetti Y., Wallace B. (2005). Applications of prism adaptation: a tutorial in theory and method. *Neuroscience & Biobehavioral Reviews*.

[43] Fernández-Ruiz J., Díaz R. (1999). Prism adaptation and aftereffect: specifying the properties of a procedural memory system. *Learning & Memory*.

[44] Spierer L., Bellmann-Thiran A., Maeder P., Murray M. M., Clarke S. (2009). Hemispheric competence for auditory spatial representation. *Brain*.

[45] Adriani M., Maeder P., Meuli R. (2003). Sound recognition and localization in man: specialized cortical networks and effects of acute circumscribed lesions. *Experimental Brain Research*.

[46] Clarke S., Thiran A. B., Maeder P. (2002). What and where in human audition: selective deficits following focal hemispheric lesions. *Experimental Brain Research*.

[47] Clarke S., Bellmann A., Meuli R. A., Assal G., Steck A. J. (2000). Auditory agnosia and auditory spatial deficits following left hemispheric lesions: evidence for distinct processing pathways. *Neuropsychologia*.

[48] Cogné M., Knebel J.-F., Klinger E. (2016). The effect of contextual auditory stimuli on virtual spatial navigation in patients with focal hemispheric lesions. *Neuropsychological Rehabilitation*.

[49] Ducommun C. Y., Michel C. M., Clarke S. (2004). Cortical motion deafness. *Neuron*.

[50] Duffour-Nikolov C., Tardif E., Maeder P. (2012). Auditory spatial deficits following hemispheric lesions: dissociation of explicit and implicit processing. *Neuropsychological Rehabilitation*.

[51] Rey B., Frischknecht R., Maeder P., Clarke S. (2007). Patterns of recovery following focal hemispheric lesions: relationship between lasting deficit and damage to specialized networks. *Restorative Neurology and Neuroscience*.

[52] Azouvi P., Bartolomeo P., Beis J.-M., Perennou D., Pradat-Diehl P., Rousseaux M. (2006). A battery of tests for the quantitative assessment of unilateral neglect. *Restorative Neurology and Neuroscience*.

[53] Knebel J.-F., Javitt D. C., Murray M. M. (2011). Impaired early visual response modulations to spatial information in chronic schizophrenia. *Psychiatry Research: Neuroimaging*.

[54] Corbetta M., Kincade J. M., Shulman G. L. (2002). Neural systems for visual orienting and their relationships to spatial working memory. *Journal of Cognitive Neuroscience*.

[55] Silver M. A., Kastner S. (2009). Topographic maps in human frontal and parietal cortex. *Trends in Cognitive Sciences*.

[56] Corbetta M., Shulman G. L. (2002). Control of goal-directed and stimulus-driven attention in the brain. *Nature Reviews Neuroscience*.

[57] Koch G., Cercignani M., Bonnì S. (2011). Asymmetry of parietal interhemispheric connections in humans. *Journal of Neuroscience*.

[58] Koch G., Oliveri M., Cheeran B. (2008). Hyperexcitability of parietal-motor functional connections in the intact left-hemisphere of patients with neglect. *Brain*.

[59] Westerhausen R., Grüner R., Specht K., Hugdahl K. (2009). Functional relevance of interindividual differences in temporal lobe callosal pathways: a DTI tractography study. *Cerebral Cortex*.

[60] Harrington I. A., Stecker G. C., Macpherson E. A., Middlebrooks J. C. (2008). Spatial sensitivity of neurons in the anterior, posterior, and primary fields of cat auditory cortex. *Hearing Research*.

[61] Stecker G. C., Harrington I. A., Middlebrooks J. C. (2005). Location coding by opponent neural populations in the auditory cortex. *PLoS Biology*.

[62] Stecker G. C., Mickey B. J., Macpherson E. A., Middlebrooks J. C. (2003). Spatial sensitivity in field PAF of cat auditory cortex. *Journal of Neurophysiology*.

[63] Woods T. M., Lopez S. E., Long J. H., Rahman J. E., Recanzone G. H. (2006). Effects of stimulus azimuth and intensity on the single-neuron activity in the auditory cortex of the alert macaque monkey. *Journal of Neurophysiology*.

[64] Recanzone G. H. (2000). Spatial processing in the auditory cortex of the macaque monkey. *Proceedings of the National Academy of Sciences*.

[65] Arnott S. R., Binns M. A., Grady C. L., Alain C. (2004). Assessing the auditory dual-pathway model in humans. *NeuroImage*.

[66] Bushara K. O., Weeks R. A., Ishii K. (1999). Modality-specific frontal and parietal areas for auditory and visual spatial localization in humans. *Nature Neuroscience*.

[67] De Santis L., Clarke S., Murray M. M. (2007). Automatic and intrinsic auditory “what”and “where”processing in humans revealed by electrical neuroimaging. *Cerebral Cortex (New York, N.Y.: 1991)*.

[68] Maeder P. P., Meuli R. A., Adriani M. (2001). Distinct pathways involved in sound recognition and localization: a human fMRI study. *NeuroImage*.

[69] Kaiser J., Lutzenberger W., Preissl H., Ackermann H., Birbaumer N. (2000). Right-hemisphere dominance for the processing of sound-source lateralization. *The Journal of Neuroscience: The Official Journal of the Society for Neuroscience*.

[70] At A., Spierer L., Clarke S. (2011). The role of the right parietal cortex in sound localization: a chronometric single pulse transcranial magnetic stimulation study. *Neuropsychologia*.

[71] Lewald J., Meister I. G., Weidemann J., Töpper R. (2004). Involvement of the superior temporal cortex and the occipital cortex in spatial hearing: evidence from repetitive transcranial magnetic stimulation. *Journal of Cognitive Neuroscience*.

[72] Cammoun L., Thiran J. P., Griffa A., Meuli R., Hagmann P., Clarke S. (2015). Intrahemispheric cortico-cortical connections of the human auditory cortex. *Brain Structure & Function*.

[73] Dietz M. J., Friston K. J., Mattingley J. B., Roepstorff A., Garrido M. I. (2014). Effective connectivity reveals right-hemisphere dominance in audiospatial perception: implications for models of spatial neglect. *Journal of Neuroscience*.

[74] Recanzone G. H. (1998). Rapidly induced auditory plasticity: the ventriloquism aftereffect. *Proceedings of the National Academy of Sciences*.

[75] Bonath B., Noesselt T., Martinez A. (2007). Neural basis of the ventriloquist illusion. *Current Biology*.

[76] Bonath B., Noesselt T., Krauel K., Tyll S., Tempelmann C., Hillyard S. A. (2014). Audio-visual synchrony modulates the ventriloquist illusion and its neural/spatial representation in the auditory cortex. *NeuroImage*.

